# Changes in Liver Steatosis Using Controlled Attenuation Parameter among Patients with Chronic Hepatitis C Infection Treated with Direct-Acting Antivirals Therapy Who Achieved Sustained Virological Response

**DOI:** 10.3390/diagnostics12030702

**Published:** 2022-03-13

**Authors:** Anca Trifan, Ermina Stratina, Adrian Rotaru, Remus Stafie, Sebastian Zenovia, Robert Nastasa, Laura Huiban, Catalin Sfarti, Camelia Cojocariu, Tudor Cuciureanu, Cristina Muzica, Stefan Chiriac, Irina Girleanu, Ana-Maria Singeap, Carol Stanciu

**Affiliations:** 1 Department of Gastroenterology, Grigore T. Popa University of Medicine and Pharmacy, 700111 Iasi, Romania; ancatrifan@yahoo.com (A.T.); adrianrotaru94@yahoo.com (A.R.); stafieremus@gmail.com (R.S.); sebastianzenovia20@gmail.com (S.Z.); huiban.laura@yahoo.com (L.H.); cvsfarti@gmail.com (C.S.); cameliacojocariu@yahoo.com (C.C.); drcuciureanutudor@gmail.com (T.C.); lungu.christina@yahoo.com (C.M.); stefannchiriac@yahoo.com (S.C.); gilda_iri25@yahoo.com (I.G.); anamaria.singeap@yahoo.com (A.-M.S.); stanciucarol@yahoo.com (C.S.); 2 Department of Gastroenterology and Hepatology, “St. Spiridon” University Hospital, 700115 Iasi, Romania

**Keywords:** liver steatosis, liver fibrosis, controlled attenuation parameter, sustained virological response at 12 weeks

## Abstract

Chronic hepatitis C virus (HCV) infection induces hepatic steatosis due to viral and host factors. However, information regarding the effects of direct-acting antivirals (DAAs) therapy on liver steatosis and fibrosis is limited. Vibration-controlled transient elastography (VCTE) with a controlled attenuation parameter (CAP) represents a non-invasive method, which has been used in the last few years for the detection of hepatic steatosis and fibrosis before and at a sustained virological response at 12 weeks (SVR12). The aim of this study was to assess the modifications of liver steatosis and fibrosis in HCV-infected patients who achieved SVR12. Consecutive patients with chronic HCV infection that were treated with DAAs in a tertiary gastroenterology center from Romania were included. Demographics, laboratory data, and VCTE evaluation were recorded in all patients. Patients with previous hepatic decompensation and those who did not achieve SVR were excluded. Two hundred and eighty patients (67.1% females) who achieved SVR12 were included. Regarding the changes in biological parameters, including liver enzymes such as alanine aminotransferase (ALT) and aspartate aminotransferase (AST), reduced to normal levels at SVR12 compared to the baseline (28.72 ± 24.71 U/L vs. 40.72 ± 27.34 U/L for ALT, *p* < 0.013 and 27.21 ± 11.15 U/L vs. 33.35 ± 23.37 U/L for AST, *p* = 0.029). On the contrary, the levels of triglycerides increased significantly from the baseline to SVR12 (124.03 ± 113.49 mg/dL to 153.78 ± 94.53, *p* = 0.004). Regarding hepatic steatosis by CAP evaluation, at SVR12, 186 (66.4%) of the individuals had a CAP score of ≥248 dB/m, an increase of 4.6% from the baseline. After viral eradication with DAAs, we observed an increase in hepatic steatosis. Hence, a long-term follow-up is mandatory to identify HCV-infected patients with hepatic steatosis post-SVR and the risk factors for more severe outcomes.

## 1. Introduction

Chronic hepatitis C virus (HCV) infection is a significant health problem affecting approximately 71 million individuals globally, causing significant morbidity and mortality rates through liver cirrhosis and hepatocellular carcinoma (HCC) [1,2]. For approximately two decades, the most used treatment of HCV infection was represented by interferon (IFN)-based regimens with a modest efficacy for achieving sustained virological response (SVR), poor tolerability, and many adverse events. Instead, the oral therapy with direct-acting antivirals (DAAs) represents an innovation for the treatment of HCV infection, with a successful viral eradication in over 95% of patients across different genotypes, offering a good safety profile with minimal side effects [3,4]. Liver fibrosis and steatosis are prognostic factors for liver disease in HCV-infected patients. Hence, SVR obtained by using DAAs therapy has been linked with liver fibrosis regression and consequently with a reduction in liver-related events such as liver decompensation and mortality [5].

The prevalence of liver steatosis among HCV-infected patients is about 55%, ranging from 40 to 86% in different genotypes [6]. Moreover, approximately 10% of patients with chronic HCV-infection have characteristics of non-alcoholic steatohepatitis. The type of hepatocyte steatosis in HCV patients depends on their HCV genotype (GNT). In patients with HCV-infection, GNT 3 is considered to be a steatogenic factor leading to “viral steatosis” caused by the cytopathic effect induced by the suppression of viral proteins [7,8]. In the case of HCV non-3 genotypes, the host factors such as age, gender, higher body mass index (BMI), or insulin resistance add on to the development of the “metabolic steatosis” [9]. Therefore, both viral and host metabolic-related factors are involved in steatosis development, and the routine evaluation of the severity of hepatic fibrosis and steatosis must be a part of the management of HCV-infected patients after the completion of DAAs therapy [10].

Recent epidemiological studies have suggested that the complex interactions between HCV and glucose metabolism lead to a higher prevalence of type 2 diabetes mellitus (T2DM) [11]. Furthermore, in patients with a higher BMI (BMI ≥ 25 kg/m^2^), the presence of chronic HCV infection increases the risk to develop T2DM by 11-fold. The main mechanism by which HCV infection induces T2DM is insulin resistance (IR). HCV infection alters the hepatocyte insulin-signaling pathways and enhances the release of proinflammatory cytokines and the serine phosphorylation of the insulin receptors, which contribute to IR [12].

Liver biopsy (LB) still remains the gold standard method for assessing hepatic fibrosis and steatosis [13,14]. However, LB is not a routinely-used technique because of several limitations such as intra- and interobserver variability, sampling errors, poor tolerability by the patient, and high cost. LB is also associated with the risk of rare but potentially life-threatening complications [15].

Vibration-controlled transient elastography (VCTE) with a controlled attenuation parameter (CAP) represents a non-invasive technique for the assessment of liver fibrosis and steatosis [16]. The advantages of this technique are represented by quickness, painfulness, and easiness to perform, with great repeatability and reproducibility, and it has been successfully implemented in clinical practice [17]. CAP is determined simultaneously with liver stiffness measurements (LSM) because it uses the same ultrasonic signal probe and measures the coefficient of attenuation of the liver at 3.5 MHz frequency [18].

Several recent studies have found a significant regression of liver fibrosis after DAA treatment, but on the other hand, data from the literature are limited and contradictory regarding steatosis [19,20]. Hence, this study evaluated changes in liver steatosis using a CAP in patients with HCV infection who achieved SVR after 12 weeks of therapy completion.

## 2. Materials and Methods

### 2.1. Patients

This prospective study enrolled 280 patients with HCV infection, who were treated with DAAs between November 2019 and November 2021 in a tertiary referral hospital in North-East Romania. Eligibility criteria were represented by: (1) age > 18 years; (2) detectable HCV RNA before treatment; (3) the achievement of a sustained virological response at 12 weeks (SVR12) after end-of treatment; (4) patients without any history of liver cancer at the baseline or during the follow-up period; (5) assessment of liver fibrosis and steatosis using VCTE at the baseline and at SVR12.

The study was performed in accordance with the principles of the Declaration of Helsinki and was approved by the Ethics Committee of our Institute. Each individual signed an informed written consent.

### 2.2. Clinical and Laboratory Assessment

Demographic and clinical details were collected including sex, age, alcohol consumption, body mass index (BMI), the presence of diabetes (taking anti-diabetic drugs or fasting glucose ≥ 126 mg/dL), and arterial hypertension (antihypertensive drugs use, systolic blood pressure ≥ 140 mmHg, or diastolic blood pressure ≥ 90 mmHg). Fasting blood tests were collected at inclusion and 12 weeks after treatment, including hemoglobin, platelet count, international normalized ratio (INR), fibrinogen, alanine aminotransaminase (ALT), aspartate aminotransferase (AST), γ-glutamyl transpeptidase (GGT), alkaline phosphatase (ALP), direct and total bilirubin (DB, TB), albumin, total proteins, urea, serum creatinine, total cholesterol, triglycerides, low-density lipoprotein (LDL-c), high-density lipoprotein (HDL-c), ferritin, c-reactive protein (CRP), α-fetoprotein, and serum HCV-RNA level.

BMI was calculated using height and weight. Weight class was defined using standard cut-offs (normal 18.5 to <25 kg/m^2^; overweight 25 to <30 kg/m^2^; obesity ≥ 30 kg/m^2^) [21]. Weight change was defined as the difference between weight at the baseline and at SVR12. The weight gain was considered as weight change ≥ 1 kg from the baseline to SVR12, and this was chosen to be clinically significant [22].

### 2.3. VCTE Examination

The patients included in our study were examined for liver fibrosis and steatosis using the FibroScan^®^ 502 touch model (Echosens, Paris, France) equipped with the M-(standard) or XL-(obese) probe by a single operator with more than 300 determinations in VCTE practice. After at least four hours of fasting, patients were placed in the supine position with the right arm in maximum abduction, leading to a greater intercostal window to the right lobe liver scanning. The examination was first performed using the M-probe with a transducer frequency of 3.5 MHz, while the XL-probe (2.5 MHz) was automatically used at machine indication if the distance between skin-to-liver capsule was higher than 25 mm. The criteria for a reliable measurement were considered to be if 10 acquisitions were made with an interquartile range divided by the median (IQR/M), which does not exceed 30%. The LSM and CAP measurement were performed at the baseline and at SVR12 [23].

In accordance with CAP measurement, which is a quantitative method and is expressed in decibel-milliwatts (dB/m) ranging from 100 to 400 dB/m, the cut of values for mild (S1), moderate (S2), and severe steatosis (S3) were as follows: ≥248 dB/m, ≥268 dB/m, and ≥280 dB/m, respectively [24]. Regarding LSM, we categorized the cut-off for staging liver fibrosis according to the Metavir scoring system: F0 (no fibrosis) ≤ 5.5 kPa; F1 (mild fibrosis) ≤ 6.9 kPa; F2 (significant fibrosis) ≥7.0 kPa; F3 (advanced fibrosis) ≥ 9.5 kPa; F4 (cirrhosis) ≥ 12.5 kPa. LSM results were expressed in kilopascals (kPa) ranging from 1.5 to 75 kPa [25].

### 2.4. Statistical Analysis

All data were analyzed using SPSS software (IBM SPSS Inc, Chicago, IL, USA, version 22.0). Continuous variables are expressed as median and interquartile/median range (IQR) or as mean ± standard deviation (SD). The Mann–Whitney test was performed to assess the difference in CAP values according to the weight gain at SVR. For comparing group’s variables, Student t-tests were used for continuous variables, or the Chi-squared test was used for comparing qualitative data as appropriate. Statistical significance was taken as *p* < 0.05 (two-tailed).

## 3. Results

### 3.1. Patients Characteristics

A total of 280 HCV-infected patients who fulfilled the inclusion criteria were evaluated between November 2019 to November 2021. At the baseline, the mean age was 59.91 ± 12.185 years, mostly females (67.1%), and BMI was 26.96 ± 4.15 kg/m^2^. Before the eradication of HCV infection, 173 patients (61.78%) had different stages of liver steatosis. All patients were treated with DAAs and obtained SVR. No patients had previous DAA treatment. Regarding the oral therapy used for the treatment of HCV infection, ombitasvir/paritaprevir/ritonavir + dasabuvir were used in 66.4% of patients and sofosbuvir/ledipasvir in 33.6% of patients.

### 3.2. Changes in CAP and LSM

**Figure 1:**
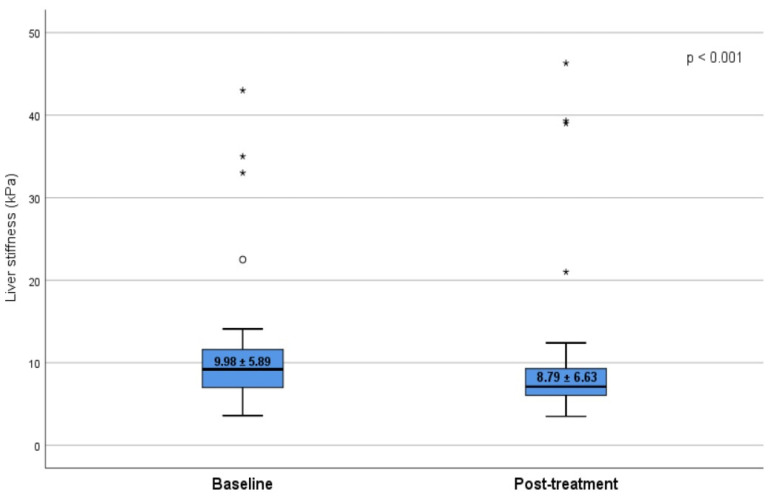
Changes in the LSM values after DAAs therapy. The bottom and the top of each box represent the 25th and 75th percentiles, while the lines through the box indicate mean value. The errors bars indicate the 10th and 90th percentiles, excluding asterisk (*). LSM, liver stiffness measurement.

**Figure 2:**
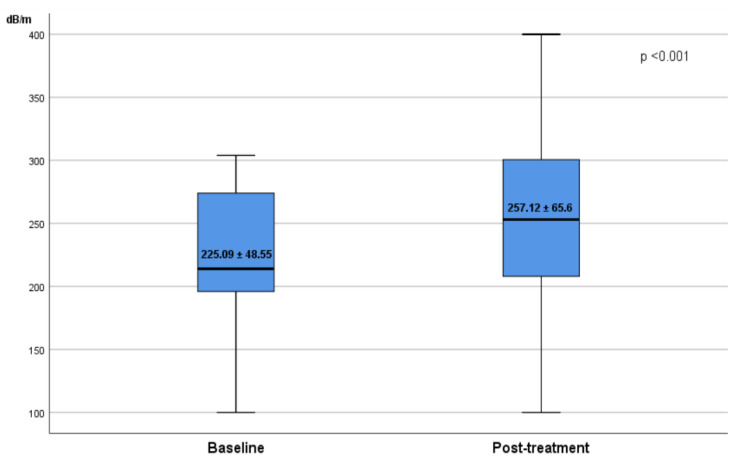
Alteration of the CAP level before and after HCV eradication.

The median (IQR) LSM and CAP values before treatment were 9.9 ± 3.7 kPa and 225 ± 48.28 dB/m, respectively. According to the baseline LSM values, the grades of liver fibrosis were as follows: F0 in 21.8% of patients, F1 in 15% of patients, F2 in 20.7% of patients, F3 in 13.9% of patients, and F4 in 28.6% of patients. Regarding the steatosis degree at the baseline, the results were as follows: S0 in 38.2% of patients, S1 in 16.5% of patients, S2 in 20.7% of patients, and S3 in 24.6% of patients (Table 1). Moreover, in the overall cohort, the LSM decreased from baseline value 9.9 ± 5.89 (5.2–15.4) kPa to 8.79 ± 6.63 (3.8–12.1) kPa at SVR12, a decline that is considered significant. The LSM reduction was observed in all drug regimens (Figure 1). The median value of CAP increased after HCV eradication. One hundred eighty-six patients (66.4%) had steatosis, with a mean CAP score of 257 ± 65.49 compared to a mean CAP score of 225 ± 48.25 dB/m at the baseline (*p* < 0.0001) (Figure 2).

### 3.3. Changes in Clinical and Biological Parameters

The factors that were associated with an increase in CAP values from the baseline to SVR are represented by impaired fasting glucose (fasting plasma glucose at baseline 106.37 ±18.86 mg/dL vs. 114.96 ± 47.19 mg/dl at SVR, *p* < 0.024), triglycerides (124.03 ± 113.49 mg/dL vs. 153.78 ± 94.53 mg/dL, *p* < 0.004), cholesterol (191.61 ± 67.19 mg/dL vs. 216.52 ± 50.85, *p* = 0.031), and higher body mass index (26.96 ± 4.15 kg/m^2^ vs. 27.87 ± 4.23 kg/m^2^). After treatment, ALT and AST decreased to normal levels at SVR12 compared to the baseline (28.72 ± 24.71 U/L vs. 40.72 ± 27.34 U/L for ALT, *p* < 0.013 and 27.21 ± 11.15 U/L vs. 33.35 ± 23.37 U/L for AST, *p* < 0.029). The BMI increased at SVR12 compared to the baseline (27.87 ± 4.23 kg/m2 vs. 26.96 ± 4.15 kg/m^2^, *p* = 0.042) (Table 1). In addition, 156 patients (56%) had impaired fasting glucose after the completion of treatment with DAAs, and 34 (22%) patients had a BMI ≥ 25 kg/m^2^. Regarding the patients with impaired fasting glucose, 125 (80%) of them had CAP values ≥ 248 dB/m.

### 3.4. Clinical and Biological Parameters in Patients with Liver Steatosis at SVR12 and in Those with No Hepatic Steatosis at SVR12

**Figure 3:**
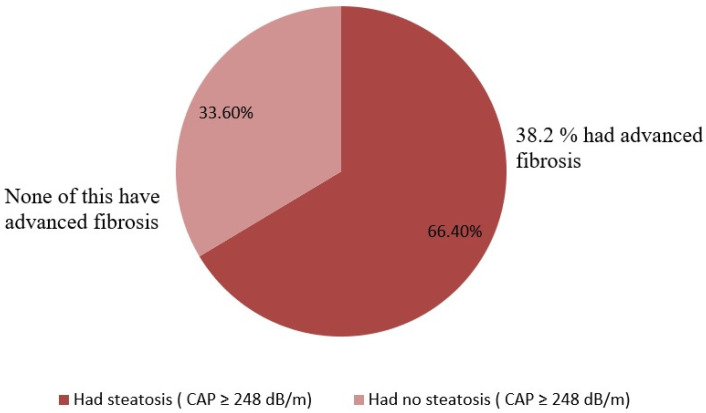
The prevalence of liver steatosis in patients with HCV infection at SVR12.

The patients with steatosis had higher LSMs (7.1 ± 1.5 kPa; SVR12- 8.3 ± 3.8 kPa, *p* = 0.038), while patients without steatosis did not have higher LSMs (7.5 ± 1.4; SVR12 5.5 ± 1.2 kPa, *p* < 0.0001) (Table 2). Thus, 38.2% of the patients with steatosis had advanced fibrosis (F3), while patients without steatosis did not have advanced fibrosis (Figure 3). At SVR12, both weight and BMI increased in patients with steatosis (73.1 ± 11.21 kg vs. 85.05 ± 10.4 kg, *p* = 0.006, respectively, 25.19 ± 5.17 kg/m^2^ vs. 29.23 ± 4.51 kg/m^2^, *p* = 0.003). The levels of transaminases decreased in patients with or without steatosis, including ALT (45.6 ± 50.8 U/L to 20.3 ± 4.5 U/L in patients with steatosis, *p* < 0.0001, and 57.78 ± 42.1 U/L to 19.4 ± 12.5 U/L in patients without steatosis, *p* < 0.0001, respectively); AST (36.3 ± 35.6 U/L to 18.3 ± 4.4 U/L, *p* < 0.0001, and 71.3 ± 58.7 U/L to 21.8 ± 10.8 U/L, *p* < 0.0001, respectively); and alkaline phosphatase (81.4 ± 42.1 U/L to 60.8 ± 24.5 U/L, *p* = 0.01, and 79.5 ± 22.8 U/L to 61.2 ± 18.4 U/L, *p* = 0.05).

### 3.5. Factors Associated with Increased CAP

**Figure 4:**
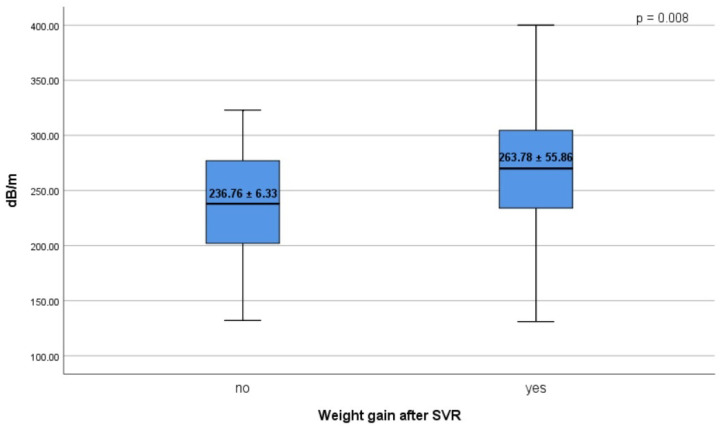
Weight gain after SVR, stratified by CAP values.

We performed a univariate linear regression analysis to identify the risk factors associated with a CAP increase at SVR12, after which only those with a significant *p* value were included in the multivariate regression analysis (Table 3). In a univariate regression model, fasting plasma glucose, increased cholesterol and triglycerides levels, and a higher BMI were significantly associated with an increase in CAP values between the baseline and 12 weeks after completion of DAAs therapy. Multivariate linear regression analysis showed that BMI (β = 0.328, *p* < 0.001) and triglycerides (β = 0.148, *p* = 0.017) were independent risk factors associated with the CAP score in all patients. Furthermore, we noticed that patients with significant weight gain at SVR12 had higher CAP values than patients without weight gain (CAP = 263.78 ± 55.86 dB/m, *p* = 0.008 vs. CAP = 236.76 ± 6.33) (Figure 4).

## 4. Discussion

HCV infection and liver steatosis are closely associated. Furthermore, the HCV-infected patients with superimposed conditions such as metabolic syndrome and obesity should be evaluated for liver steatosis after SVR [26]. In several studies, the hepatic steatosis in HCV patients before SVR was assessed by liver biopsy, and 40–80% of cases had liver steatosis [27,28]. In our study, the prevalence of liver steatosis in patients who achieved SVR with DAAs was 66,42%, independent of the type of regimen used. In contrast, patients had normal levels of liver enzymes in the presence of steatosis, and this should be of concern to clinicians.

The American Association of the Study of Liver Diseases (AASLD) recommends the assessment for modifiable risk factors of liver injury, such as liver steatosis and diabetes mellitus, and to continue disease-specific management to optimize weight loss and glycemic control in those who achieved SVR [29]. Furthermore, an abnormal BMI and insulin resistance (IR) at the baseline are important risk factors for liver steatosis and weight gain after DAAs treatment. In patients who obtained SVR, the bidirectional relationship between weight gain and the development of diabetes causes the progression of hepatic steatosis and fibrosis [30].

In our study, clearance of HCV was not associated with an important improvement in glycemic control, with 156 patients (56%) having impaired fasting glucose after the completion of treatment with DAAs. Thirty-four (22%) of them also had a significant weight gain, suggesting that high glycemic values were due to the improvement of the quality of life and the elevation of the levels of hunger-inducing hormones in those who achieved SVR. Moreover, a CAP score ≥ 248 dB/m was found in 80% of patients with impaired fasting glucose. In addition, upon multivariate analysis, we found that BMI (β = 0.328, *p* < 0.001) and triglycerides (β = 0.148, *p* = 0.017) were independent risk factors associated with an increased CAP score. Similar results were observed by Azad et al., who noted a higher mean weight gain in diabetic patients compared to non-diabetics treated with sofosbuvir + ledipasvir [31]. Moreover, Strauhs-Nitsch et al. also demonstrated in a recent cohort study that achieving SVR after DAAs did not improve IR after one-year by using homeostatic model assessment for insulin resistance (HOMA-IR) [32]. In contrast, Ciancio et al. conducted a prospective case-control study of 122 patients and showed that viral eradication using DAAs is associated with reduced fasting plasma glucose and the need for antidiabetic therapy [33].

We observed a significant increase in BMI at SVR12 (26.96 ± 4.15 kg/m^2^ vs. 27.87 ± 4.23 kg/m), while we could also suggest that excessive calorie intake may be responsible for the liver steatosis. Do et al. had similar findings in a large cohort of 11.,469 patients and reported weight gain in 2.293 (20%) patients after DAAs treatment who achieved SVR [34]. Another study by Schlevogt et al. showed that 44% of treated subjects had weight gain at their long-term follow-up after SVR [35].

HCV infection promotes chronic systemic inflammation, reduces quality of life, and is frequently associated with depression and fatigue, with all of this leading to weight loss [36]. Other factors that may cause weight loss are represented by protein-calorie malnutrition and sarcopenia. After SVR, the chronic inflammation cycle is disrupted and there is a major improvement in liver anabolism, inducing an increased nutritional intake. These changes will finally determine IR and higher fasting glucose levels [37].

In HCV-infected patients, viral- and host-mediated factors contribute to the progress of liver steatosis. The prevalence of liver steatosis also has an important dependence on genotype. Thus, infection with GNT 3 is frequently associated with the development of HCC, liver fibrosis, and lower SVR rates with direct-acting antivirals. However, in patients with GNT 1b of HCV infection, poorer clinical outcomes and metabolic steatosis are expected [38]. In Romania, the prevalence of GNT 1b is 99.6% in HCV patients [39].

A prospective study shows that magnetic resonance (MR)-based techniques are superior to VCTE for detecting hepatic steatosis and fibrosis in patients with HCV infection. MR-based techniques have a high sensibility for hepatic steatosis detection but are not suitable as point-of-care methods due to high costs [40]. VCTE has multiple advantages, including better patient acceptability, easiness to perform, and a more affordable price than MR [26]. On the other hand, abdominal ultrasound (US) is used as a first-line assessment for the screening of fatty liver because of low cost and easy accessibility, but it is imprecise in measuring the extent of steatosis in those with morbid obesity, or in those with less than 20% of liver fat accumulation and is dependent by operator [41]. In comparison with US, CAP is quantitative tool for assessing liver steatosis with higher sensitivity and specificity. CAP numerical values measurements also correlate with the histological degree of steatosis, being a quick and easy method to perform [42,43].

Nourredin et al. found, in a prospective study consisting of 101 patients with HCV infection who have obtained SVR with DAAs therapy, that liver steatosis is a common finding post-SVR surveillance. A total of 48 patients had liver steatosis at 47 weeks after the end of treatment with a mean CAP score of 296.31 ± 37.4 dB/m [44]. In line with these results, our study showed that 186 patients (66.4%) had liver steatosis at SVR12, with a mean CAP score of 257 ± 65.49 dB/m, and these results show an increase of 4.6% from the baseline. Additionally, the patients with liver steatosis had a higher fibrosis score than the patients without liver steatosis at SVR12 (8.3 vs. 5.5 kPa). Similar results were found by Ogasawara et al. who conducted a study in Japan; they analyzed a similar group, consisting of 214 patients with HCV infection treated with DAAs, and they concluded that liver steatosis increased significantly after SVR [45]. In another prospective study, Rout et al. had similar findings, with an increase in the CAP score in a cohort of 408 HCV-infected patients who achieved SVR after therapy with DAAs [46].

In contrast, in a retrospective study conducted by Shimizu et al., which included 70 patients with chronic HCV infection who achieved SVR at 12 weeks using DAAs therapy, they reported a decrease in CAP values at SVR12. Moreover, the decrease was higher among patients with S3 compared with those with S1 and S2 degrees of liver steatosis [47].

Recent studies among predominantly genotype 1 HCV-infected patients suggest an effect of DAAs therapy on cholesterol and triglyceride levels [48,49]. Thus, DAAs therapy induce elevated triglycerides and cholesterol levels [50,51]. In line with these reports, we observed a significant increase in triglyceride levels (124.03 ± 113.49 mg/dL vs. 153.78 ± 94.53 mg/dL) and in cholesterol levels (191.61 ± 67.19 mg/dL vs. 216.52 ± 50.85), respectively. These results can explain the higher values of CAP measurements and need to be evaluated furthermore. Moreover, we documented a significant increase in the body mass index at SVR12 (26.96 ± 4.15 kg/m^2^ vs. 27.87 ± 4.23 kg/m^2^), and that fact could suggest that an excessive calorie intake may be responsible for the liver steatosis.

One strength of our study consists of assessing a detailed metabolic profile that is recognized as a cause for lipid deposition in the liver, especially in patients with HCV infection. Indeed, our study also had a few limitations. The first limitation is that the median time interval for the follow-up in our study was 12 weeks after treatment completion. Another limitation is represented by the measurements of LSM and CAP at SVR12, with no further follow-up. For assessing the progression of liver steatosis and fibrosis in HCV-infected patients, lengthier studies are needed [52]. The absence of histological assessment should be mentioned as a limitation. Finally, we did not accumulate data about the patient’s lifestyles, regarding dietary regime, or the frequency of physical activity.

## 5. Conclusions

In light of the latest evidence, it is clear that in the HCV-infected patients treated with DAAs therapy who achieved SVR, the liver steatosis increased, and fibrosis score decreased. In our study, 66.4% of HCV patients had hepatic steatosis at SVR12. Moreover, 38.2% of them had at least advanced fibrosis (≥F3), despite normal levels of liver enzymes. In patients who obtained SVR, the AASLD recommends counseling on lifestyle changes to prevent weight gain and to improve glycemic control [29]. Our results suggest that SVR is an opportune time to assess the importance of weight gain in the long-term as a metabolic risk factor for liver outcomes. At SVR12, the assessment of steatosis and fibrosis in those with a BMI ≥ 25 kg/m^2^ or other risk factors related with HCV-infection is also definitely warranted. Future studies are needed to clarify the importance of a long-term assessment of liver steatosis and the outcomes associated with weight gain post-SVR, as well as the role of clinical strategies to prevent weight gain after DAAs therapy.

## Figures and Tables

**Table 1:** Patient characteristics.

	Overall Cohort *n* (%)	Characteristics Baseline	Characteristics SVR12	*p*-Value
Variable	Overall (*n* = 280)			
Gender (female), *n* (%)	188 (67.1)			
Age, yr.	59.91 ± 12.185			
BMI (kg/m^2^)	27.13 ± 3.62	26.96 ± 4.15	27.87 ± 4.23	0.042
HGB (g/dl)	13.23 ± 1.67	13.04 ± 1.48	13.32 ± 1.56	0.651
Platelet count (G/L)	192.18 ± 66.18	188.42 ± 71.39	193.53 ± 68.23	0.798
ALT (IU/L)	30.24 ± 26.76	40.72 ± 27.34	28.72 ± 24.71	0.013
AST (IU/L)	31.77 ± 22.61	33.35 ± 23.37	27.21 ± 11.15	0.029
GGT (IU/L)	41.89 ± 48.91	40.64 ± 31.07	43.33 ± 38.03	0.237
ALP (IU/L)	80.70 ± 36.92	76.67 ± 30.65	79.37 ± 35.01	0.709
Total bilirubin (mg/dL)	0.72 ± 0.68	0.75 ± 0.39	0.69 ± 0.38	0.465
Albumin (g/dL)	4.56 ± 0.38	4.53 ± 0.44	4.57 ± 0.36	0.559
Creatinine (mg/dL)	0.73 ± 0.13	0.71 ± 0.13	0.73 ± 0.13	0.505
Urea (mg/dL)	36.56 ± 10.81	34.40 ± 37.08	37.08 ± 11.22	0.188
Fasting glucose (mg/dL)	111.37 ± 43.77	106.37 ± 18.86	114.96 ± 47.19	0.024
Total cholesterol (mg/dL)	211.68 ± 55.04	191.61 ± 67.19	216.52 ± 50.85	0.031
Triglycerides (mg/dL)	148 ± 98.78	124.03 ± 113.49	153.78 ± 94.53	0.004
CAP dB/m	293 (245.5–339)	225 ± 48.28	257 ± 65.49	<0.001
CAP ≥ 248 dB/m *n* (%)		173 (61.8%)	186 (66.4%)	
Steatosis degree				
S0 *n*(%)		107(38.2%)	94 (33.6%)	
S1 *n*(%)		46 (16.5%)	56 (20%)	
S2 *n* (%)		58 (20.7%)	62 (22.1%)	
S3 *n* (%)		69 (24.6%)	68 (24.3%)	
Fibrosis score (mean ± SD)		9.98 ± 5.89	8.79 ± 6.63	0.019
Fibrosis stages				
F0 *n* (%)		61 (21.8%)	72 (25.7%)	
F1 *n* (%)		42 (15%)	54 (19.3%)	
F2 *n* (%)		58 (20.7%)	47 (16.8%)	
F3 *n* (%)		39 (13.9%)	44 (15.7%)	
F4 *n* (%)		80 (28.6%)	63 (22.5%)	

BMI, body mass index; AST, aspartate aminotransferase; ALT, alanine aminotransferase; GGT, gamma-glutamyl transferase; ALP, alkaline phosphatase.

**Table 2:** Comparison of patients with and without steatosis at baseline vs. SVR12.

	Patients without Steatosis (*n* = 94)			Patients with Steatosis (*n* = 186)		
	Baseline	SVR12	*p*-Value	Baseline	SVR12	*p*-Value
Body mass index (kg/m^2^)	25.3 ± 5.0	26.1 ± 5.9	NS	25.19 ± 5.17	28.15 ± 4.51	0.003
Weight (Kg)	72.4 ± 4.53	73.4 ± 3.27	NS	73.1 ± 11.21	85.05 ± 10.4	0.006
Laboratory panel (mean ± SD)						
HCV viral load log10 IU/mL	7.1 ± 1.4	0.0 ± 0.0	0.0001	7.1 ± 1.4	0.0 ± 0.0	0.0001
AST (U/L)	71.3 ± 58.7	21.8 ± 10.8	0.0001	36.3 ± 35.6	18.3 ± 4.4	0.0001
ALT (U/L)	57.78 ± 42.1	19.4 ± 12.5	0.0001	45.6 ± 50.8	20.3 ± 4.5	0.0001
Alkaline phosphatase (U/L)	79.5 ± 22.8	61.2 ± 18.4	0.05	81.4 ± 42.1	60.8 ± 24.5	0.01
Fibrosis score (kPa)	7.5 ± 1.4	5.5 ± 1.2	0.0001	7.5 ± 1.5	8.3 ± 3.8	0.038

HCV, hepatitis C virus; AST, aspartate aminotransferase; ALT, alanine aminotransferase; kPa, kilopascal.

**Table 3:** Univariate and multivariate analyses of factors associated with increased CAP values.

Variable	Univariate		Multivariate	
	β	*p*	β	*p*
Age	0.126	0.287		
Gender	0.07	0.912		
BMI (kg/m^2^)	0.561	<0.001	0.328	<0.001
HGB (g/dL)	0.143	0.728		
Platelet count (G/L)	−0.072	0.896		
ALT (IU/L)	−0.15	0.872		
AST (IU/L)	−0.218	0.726		
GGT (IU/L)	0.182	0.787		
ALP (IU/L)	−0.139	0.639		
Fasting glucose (mg/dL)	0.299	0.041	0.187	0.056
Creatinine (mg/dL)	0.331	0.648		
Urea (mg/dL)	−0.013	0.627		
Total cholesterol (mg/dL)	0.310	0.008	0.108	0.052
Triglicerides (mg/dL)	0.426	<0.001	0.148	0.017
Albumin (g/dL)	−8.44	0.711		
Total bilirubin (mg/dL)	−0.23	0.580		
Baseline CAP	−0.594	<0.001	−0.596	<0.001

## Data Availability

The data presented in this study are available on request from the corresponding author. The data are not publicly available because they are property of the Institute of Gastroenterology and Hepatology, Iasi, Romania.
